# Sumatriptan poisoning and its clinical presentation in humans: A case report

**DOI:** 10.1002/ccr3.6453

**Published:** 2022-10-19

**Authors:** Peyman Erfan Talab Evini, Laya Ohadi, Farbod Amiri, Shahin Shadnia, Babak Mostafazadeh, Mehdi Forouzesh, Mohammad Javad Hedayatshodeh, Mitra Rahimi

**Affiliations:** ^1^ Toxicological Research Center, Clinical Toxicology Department, Loghman Hakim Hospital, School of Medicine Shahid Beheshti University of Medical Sciences Tehran Iran; ^2^ Shahid Beheshti University of Medical Sciences Tehran Iran; ^3^ Iranian Legal Medicine Research Center Tehran Iran

**Keywords:** complication, nephritic syndrome, poisoning, sumatriptan

## Abstract

This case report displays some of the possible complications of sumatriptan poisoning, including nephritic syndrome.

## Abstract 2

This study aims to demonstrate some of the adverse effects related to sumatriptan toxicity; a drug is used to treat migraine. Other studies are required to reveal these side effects, especially for patients with underlying diseases such as chronic kidney disease.

## 1 INTRODUCTION

One of the most prevalent and socioeconomically impactful disabling primary headache is migraine, and its acute daily attacks can be treated by prescription of sumatriptan orally or subcutaneously.^1^, ^2^

This potent drug acts on 5‐HT1 receptors, leading to vasoconstriction. Like other medications, it has side effects like tachycardia, transient hypertension, behavioral instability, facial edema, angle‐closure glaucoma, cardiac arrhythmias, abdominal pain, bloody diarrhea because of vascular ischemia, splenic infarction, hepatotoxicity, and Raynaud's syndrome.^3^, ^4^, ^5^, ^6^

Previously, Owen et al. revealed side effects of sumatriptan poisoning in rodents and dogs. Moreover, Mobasheran et al. demonstrated renal injury by activating nitric oxide synthase in rats induced by sumatriptan, but there is no available information in humans.^3^, ^5^, ^7^ This case report presents a case with sumatriptan poisoning who had complications including nephritic syndrome.

## 2 CASE PRESENTATION

In February 2022, a 20‐year‐old bodybuilder man presented to the emergency department with unusual sweating, agitation, hallucination, and urine and fecal incontinency. He was admitted to the toxicology ward due to the injection of 30 vials of sumatriptan (each vial is 6 mg/0.5 ml solution) with the intention of committing suicide 2 days before his admission. He had no complaints, such as respiratory difficulties or chest pain.

Past medical and habitual history for any drug or substances were negative. At admitted time, vital signs demonstrated high blood pressure (150/90 mm of mercury) and tachycardia (126 per minute). On the contrary, respiratory rate (18 per minute), temperature (37.1°C), and oxygen saturation (99%) were normal. Moreover, the initial Glasgow coma scale (GCS) was 15/15.

According to the protocol, primary conservative treatment was started for this patient. After a few hours, he became unconscious (GCS = 9/15) and was intubated.

Laboratory findings displayed hypernatremia and increased creatinine, liver function markers, creatine phosphokinase (CPK), lactate dehydrogenase (LDH), and D‐dimer level. Qualitative measurement of cardiac biomarkers such as troponin was also positive in this patient. Besides, hematuria (3+) and proteinuria (2+) were detected in the urine analysis but his urine drug test was negative for morphine, tramadol, methadone, and tetrahydrocannabinol, amphetamine, and methamphetamine. Laboratory tests are described in Table 1.

**TABLE 1:** Laboratory findings at admitted time

Markers	Value w/Units	Normal range
Urea	3.3 mmol/L	2.8–8.1
Creatinine	**1.7 mg/dl**	0.7–1.3
Sodium	**153 mmol/L**	136–145
Potassium	4.5 mmol/L	3.5–5.1
White blood cell count	10 × 10^3^/ul	4.0–10.0
Hemoglobin	15 g/dl	13.2–16.6
Platelet count	321 × 10^3^/ul	150–400
Calcium	9.1 mg/dl	8.6–10.3
Magnesium	1.05 mmol/L	0.66–1.07
Albumin	4.7 g/dl	3.4–5.4
Aspartate aminotransferase	**157 U/L**	10–40
Alanine aminotransferase	**359 U/L**	7–56
Alkaline phosphatase	**219 U/L**	30–120
Creatine phosphokinase	**2620 U/L**	39–308
Lactate dehydrogenase	**1448 U/L**	140–280
PH	7.39	7.35–7.45
PCO2	44.8 mmHg	35–45
HCO3	27.4 mEq/L	22–28
PT	13.3 s	11–13.5
PTT	26.7 s	25–35
INR	1.1	<1.1

Bold values that were presented above are out of normal range.

Additionally, normal sinus rhythm was detected in his electrocardiogram (ECG). Full cardiac monitoring and serial ECG and troponin were performed for him because of positive troponin. Computed tomography (CT) angiography was requested because pulmonary embolism (PE) was suspected but was not performed due to hemodynamic instability. Due to the high suspicion of PE according to the patient's condition including bedridden and tachycardia, treatment was started immediately. Despite advanced medical treatment, the cardiopulmonary arrest happened after 6 days and the probable cause of death was ventilator‐associated pneumonia.

At autopsy, the lungs were dilated with purulent discharge, and a normal‐sized heart was observed with no apparent stenosis in coronary arteries and no fibrosis and hyperemia in myocardial sections. Besides, abnormalities were not found in the liver, spleen, or kidneys. Cerebral edema due to hypoxia was detected in the brain with normal consistency. Moreover, post‐mortem toxicological assessment had no scientific value due to more than 6 days of hospitalization.

Consequently, hypernatremia, positive troponin, elevated level of creatinine, liver enzymes, CPK and LDH, hematuria, and proteinuria were associated with sumatriptan poisoning.

## 3 DISCUSSION

Sumatriptan, a potent drug with a vasoconstriction effect prescribed in the treatment of migraine attacks, can cause minor complications, including sweating, transient hypertension, tachycardia, behavioral changes, fatigue, sleepiness, and skin reaction.^5^ Similarly, our case presented with a significant rise in blood pressure level and heart rate. Moreover, agitation and hallucination were seen in this patient. On the contrary, despite injecting 30 vials of sumatriptan, no reaction at the injection site was detected. Persistent to our study, Owen et al. demonstrated tachycardia induced by sumatriptan in animals as well.^3^ Some of these complications are related to serotonin syndrome, a drug‐induced syndrome associated with sumatriptan administration.^8^

This effective drug has major adverse effects such as hepatotoxicity by causing oxidative stress in hepatocytes.^6^ Besides, an increased level of liver enzymes in this patient was a sign of affecting the liver.

Additionally, urine and fecal incontinency were realized in this case. Another study on rabbits proved that drugs that act on 5‐HT1 receptors have regulatory action on bladder activity.^9^ On the contrary, there are no available data about this pivotal effect on humans.

Increased creatinine level was detected in this case that, previously proved by Mobasheran et al. In 2019, they performed a trial on rats that revealed the administration of sumatriptan induces renal injury by activating nitric oxide synthase and oxidative responses.^7^ In addition, bilateral renal infarction, a rare side effect, was recognized in a healthy 45‐year‐old woman after prescribing sumatriptan.^10^ Additionally, Sharma et al. reported renal cortical infarction induced by sumatriptan in a kidney allograft recipient.^11^ On the contrary, Sheibani et al. displayed that the prescription of low doses of sumatriptan in male rats has protective effects on renal injury because of its anti‐inflammatory agents.^12^

Previously, Kelly displayed that one dose of sumatriptan can cause sudden cardiac arrest because of coronary artery vasoconstriction.^13^ Despite positive troponin, and increased level of LDH and CPK, there was no positive clue about myocardial infarction in our patient according to the biopsy.

## 4 CONCLUSION

Sumatriptan, that is used to treat migraine episodes, can affect many systems in healthy humans, including cardiovascular system, causing myocardial infarction.^14^ There are some studies about the toxicity of this drug just in animals.^3^ Our case supports the novel complications of sumatriptan toxicity in humans, including urine incontinency, nephritic syndrome, and hepatotoxicity. Further clinical trials are required to reveal the complications of the sumatriptan, especially in patients with positive past medical history.

## AUTHOR CONTRIBUTIONS

PE collected the data and corrected the manuscript for its scientific basis. LO collected the data for the study and wrote the manuscript. FA wrote and corrected the manuscript for its scientific basis. SS and BM revised the manuscript for grammar and syntax mistakes. MR corrected the manuscript for its scientific basis and revised the manuscript for grammar and syntax mistakes. MF and MJH collected and wrote the autopsy part. All authors read and approved the final manuscript.

## FUNDING INFORMATION

No additional funding for the execution of the present study was received.

## CONFLICT OF INTEREST

The authors declare that they have no competing or conflict of interests.

## CONSENT

Written informed consent was obtained from the patient's family to publish this case report and any accompanying images. A copy of the written consent is available for review by the journal's Editor‐in‐Chief.

## Data Availability

The data that support the findings of this study are available on request from the corresponding author. The data are not publicly available due to privacy or ethical restrictions.
